# How to Screen Suitable Service Improve Community Health Care Services by University Students in Taiwan

**DOI:** 10.3390/ijerph17155402

**Published:** 2020-07-27

**Authors:** Guey-Shin Shyu, Shinn-Jou Lin, Wei-Ta Fang, Bai-You Cheng

**Affiliations:** 1 Department of Tourism, Tungnan University, Shenkeng District, New Taipei City 22202, Taiwan; gsshyu@mail.tnu.edu.tw; 2 Graduate Institute of Environmental Resources Management, TransWorld University, Douliu City, Yunlin County 64063, Taiwan; shinnjou@gmail.com; 3 Graduate Institute of Environmental Education, National Taiwan Normal University, Taipei City 11677, Taiwan

**Keywords:** community health services, postmodern education theories, factor analysis

## Abstract

Engaging in social contributions to enhance social participation and attending community experiential service learning or internship courses have become an essential learning experience for university students. On the basis of postmodern education theories, this study adopted images and oral accounts involving personal experiences to construct a postmodern education research scheme by using the method of collaborative ethnography. This study selected and performed the following services: filming a community documentary, administering community health dance classes, and archiving community cultural artifacts in databases. Interviews were also administered to facilitate implementation of the actual services. Community health services commonly seen in Taiwan and abroad were compiled, and the resources required for each service were examined. Subsequently, factor analysis was performed to explore the characteristic of these services in order to recommend feasible services for university students to undertake. The results indicated that the eight resources required for the 59 common community health services were (1) a designated space or venue, (2) materials, (3) monetary resources, (4) human resources, (5) expertise, (6) professional equipment, (7) patience, and (8) empathy. The results revealed three principal components, namely labor services, high-resource services, and professional services, for a total explanatory power of 67.99%; the individual explanatory power of these components accounted for 25.04%, 21.81%, and 21.15%, respectively. Next, community health care services suitable for university students to perform were selected and implemented, and these services were well received. The study results indicated that community and environmental justice can be realized by identifying with the value of community health services and promoting postmodern education theories and social norms. The research results are suitable for implementation after the COVID-19 pandemic.

## 1. Introduction

The Taiwanese government proposed the concept of comprehensive community development in 1993, aiming to establish community culture, cohere community consensus, and construct the concept of community life to formulate new thinking and policies for cultural administration [1]. This policy-based term originated from the goal of integrating the five major aspects of community development, namely people, culture, land, landscape, and production. In fact, this concept pertains to the movement of transforming community culture [2,3,4,5]. In other words, community residents voluntarily participate in related activities, exercise their creativity, and manage community affairs comprehensively to build a cultural scene of their own community. Many sociologists have emphasized that community affairs should be managed by local residents, who then combine community resources and reach consensus through public discussion to determine community development collaboratively. In short, handling community affairs involves bottom-up process, public participation, and local autonomy [6,7,8,9,10].

Regarding the implications of community, we observed value transformation in community cases that arises from changes in a modernized lifestyle [11,12]. Community development involves sociology, psychology, and cultural anthropology; hence, community-related topics are complex issues that cannot be solved simply through scientific management [13]. In other words, adequate community management does not accentuate verifying quantitative hypotheses; instead, it regards research participants as a being of mutual subjectivity, replacing mechanics-based Cartesian perspectives of instrumental rationality with an organic community worldview. In the past, modernism was represented by Cartesian instrumental rationality and Newtonian mechanics and involved a reductionistic process to understand management tools [14,15,16]. Contemporary society has entered a period of pluralistic postmodernism when modernism is overloaded with data such that rational thinking cannot guarantee effective management strategies [4]. In the postmodern era, people generally value the opinions of the disadvantaged in communities, respect different cultures and ethnic groups, and recognize the value and status of dissenting opinions [17]. Postmodernism mostly adopts a pluralistic argument that enables humans to learn to respect the differences between each other [15]. Postmodernists value interpersonal differences, oppose the objectivity and universality of knowledge emphasized in modernism, adopt small narratives for thinking, and view things from a pluralistic perspective [16]. Humans do not follow the Western scientific trajectory of development in building their societies; instead, they start to identify with their own ethnic groups and cultural features, as well as fight for their own rights. Peters and Waterman [18] argued that postmodern management should adopt a Back to Basics approach to conducting reformation [18]. The art of management should permeate every detail of society, using symbols such as consciousness, emblem, drama, vision, and love to thoroughly subvert the strict system, control, and structure of modernist management [19,20,21,22].

Community development is essentially a spontaneous activity of community residents. However, most public topics must be handled by public authorities or financially supported by the public sector. To support the particular requirements of community development, governments organize specific incentives and grants to enhance training programs and foster public and community awareness. Because community development differs from other policies in implementation methods, the Council for Cultural Affairs (now the Ministry of Culture) under the Executive Yuan proposed the concept of comprehensive community development and launched multiple grant programs. Accordingly, Taiwan’s community cooperation policies gradually extended from the Council for Cultural Affairs to the other central ministries [23].

In 2005, the Taiwanese government proposed the Six-Star Plan for a Healthy Community to increase the range of community development and government participation, implement the bottom-up approach, and improve public participation through comprehensively promoting the plan, training community development talents, and building community databases [1]. The six major aspects of the plan and their implications are as follows: (a) humanistic education: develop community awareness, strengthen the operation of community organizations, and implement community lifelong learning; (b) industrial development: promote industrial transformation and upgrading and facilitate local job opportunities; (c) social welfare and health care: develop community health care services and community health; (d) community policing: build a community security system, implement a community disaster prevention system, and develop a domestic violence prevention system; (e) environmental landscape: develop community styles and facilities as well as repurpose community space; and (f) environmental and ecological protection: promote community cleaning and improve ecological conservation. These are the principles for implementing this policy [1].

After 20 years, ideals regarding the participation and autonomous management of communities are no longer limited to the comprehensive community development policies [23]. Programs implemented by departments related to environmental protection, internal affairs, agriculture, culture, policing, economy, commerce, health, and social affairs also focus on community residents to facilitate bottom-up operation, promote spontaneity, as well as transform communities and create local atmospheres according to residents’ actual needs [24,25,26,27,28].

The aforementioned literature review indicates that postmodern education management is characterized by anti-rationalism, anti-collectivism, cultural pluralism, and the use of digital images and Internet information for management [29,30,31,32]. In discourses related to the use of digital images and internet information, community management involves records compiled through photo-voice and photo elicitation. Regardless of their level of visual and auditory aesthetics and whether the artistry of the recorded experience is accentuated, these records provide information of static material culture and dynamic spoken arts and performing arts in community life that can be examined through content analysis. Therefore, we applied the visual and auditory communication contexts of phenomenology and incorporated other phenomenological elements including on-site reading, observation, listening, conversation, reflection, and introspection [32]. How to return to the ontology of education and body, extract knowledge from the direct intuition and a priori nature of phenomenology, as well as develop a sense of love, respect, and appreciation in and incorporate self-talk into community activity observation are crucial topics for postmodern education and community studies [29,30,31,32].

In recent years, the reduced costs of video and image recording have enabled it to become a prevalent method to facilitate community development and retain community records. Local government has provided filming-related professional training courses to implement a bottom-up autonomous management scheme in communities, thereby highlighting the self-worth of the communities. Therefore, training courses for creating community documentaries have been provided. This enables community groups to develop autonomous media and express their civil power [33,34].

This study proposed an analysis process that involved recording dialogues and images and performing statistics commonly used in social science to examine the target plans and provide information for decision making. The purposes of this study had three topics: (1) summarizing the main types of community health services; (2) recommending that university students can operate these services; and (3) adding actual implementation and recording the final results from university students’ social works.

## 2. Materials and Methods

### 2.1. Research Target: Lunfeng Community at Douliu City in Central Taiwan

The target community is located in southern Douliu city in center Taiwan. Several traditional Chinese three-sided courtyard houses built in the 1900s and a sugar factory area developed during Japanese colonization still remain in the northern part of the community. Because the community boasts ample sunshine and plenty of water resources, Japanese colonizers built a sugar factory here, and the sugar refining industry once thrived in the area. After the industry declined, the area became famous for producing fruits such as pomelo and orange. The target community has approximately 2912 residents and 1107 households (Table 1). Its main agricultural products include honey tangerine, orange, pomelo, and Taiwan Giant Bamboo (*Dendrocalamus latiflorus*) shoot.

**Figure 1:**
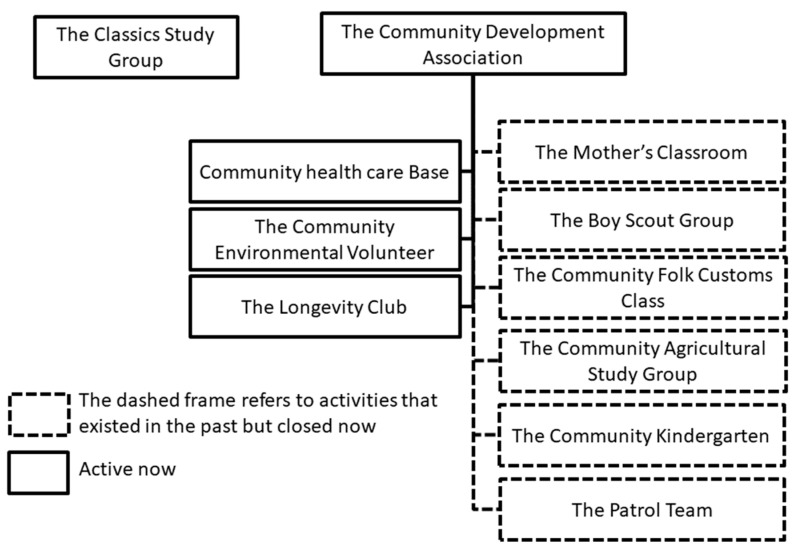
Organization overview of the target community.

Figure 1 presents the organization overview of the target community, revealing that most of the counseling organizations established under government support have ceased to operate. Currently, the Community Health Care Base and the Community Environmental Volunteer Team are the major and most active organizations that remain operational. In addition, the Longevity Club regularly organizes trips and meal gatherings for older residents in the community. Although these activities are held occasionally, the organization has a steady operation procedure.

Since there had occurred a community-based health care operated by government departments, there could be detected social development associations to run this business. For some reasons, community activity centers often have only detected resident gatherings. Our research team, therefore, usually has been hired four university students who have been involved in the community for a long stay. The internship period of the students has accounted for about one year, and often every now and then another student group could replace their duties after one year. When they were running some activities on holidays, other interns could be involved in supporting up to more than fifteen volunteer students.

The Community Health Care Base was formally established in 2015 and holds activities including health-promoting and care visits, phone greetings, regular Monday gatherings (providing blood pressure measurement services), karaoke, exercises (for promoting blood circulation), and health-promoting courses for improving older-adult services. These activities facilitate the eliciting of a sense of cohesion between the residents. The organizations of the community are listed as follows:

(1) The Community Environmental Volunteer Team cooperates with the Irrigation Association to maintain community environment and reconstruct landscapes. This team is currently one of the most active organizations in the community.
(2) The Longevity Club is an organization for older community residents to interact with each other. It organizes one trip and the Double Ninth Festival annually.
(3) The Classics Study Group was established privately with a focus on Yiguandao scriptures.
(4) The Mother’s Classroom used to offer art courses but is currently closed due to teacher shortage.
(5) The Boy Scout Group was established according to the education act promulgated by the provincial government of Taiwan and is disbanded now.
(6) The Community Folk Customs Class shut down due to teacher shortage.
(7) The Community Agricultural Study Group was established jointly with the Council of Agriculture and the 4-H Club but has terminated now.
(8) The Community Kindergarten has terminated.
(9) The Patrol Team was established in 2006 and disbanded after 2008.

### 2.2. Multivariate Factor Analysis

The purpose of factor analysis is to define latent constructs. Because latent factors involve concepts such as natural backgrounds, social justice, and values that cannot be measured directly, factor analysis can be performed to explore the structural components of such concepts, define the various constructs related to such concepts, and determine the variables associated with each construct [35,36].

Factor analysis does not differentiate dependent variables from independent variables; instead, it analyzes the relationships between all variables and utilizes their correlations to maximize the variance of all the variables. Therefore, after the structural components of the data are determined, factor analysis is usually used to summarize and reduce data. Relational concepts can be derived after the variables are examined through factor analysis. These concepts summarize all the variables without losing excessive information and become the so-called constructs after being named appropriately [34,35,36].

Factor analysis enables data reduction to select representative variables that retain most of the explanatory power of the original variables, in addition to maintaining the original data structure [35]. Moreover, because the extracted principal factors do not exist in the original data but serve as new variable synthesized through examining the data structure, they can be used to represent latent factors that cannot be categorized directly but have significant influences. This enables complementing analysis items that are left out or unclassifiable. This procedure is known as exploratory factor analysis [36].

The mathematical model of factor analysis is the Equation (1). There n denotes the number of variable (*X_1_*, *X_2_*, … *X_n_*), and m denotes the number of underlying factors (*F_1_*, *F_2_*, … *F_m_*).

*Xi* is the variable represented in latent factors.

This model assumes that there are m underlying factors whereby each observed variable is a linear function of these factors together with a residual variate.

This model intends to reproduce the maximum correlations.

(1) $$X_{i}=a_{i1}F_{1}+a_{i2}F_{2}+\cdots a_{im}F_{m}e_{i}$$

where *i* = 1, 2, …, *n*.

The factor loadings are $a_{i1}$, $a_{i2}$, $a_{im}$, which denotes that $a_{i1}$ is the factor loading of *i*th variable on the 1st factor. The specific or unique factor is denoted by $e_{i}$.

The factor loadings were how much the variable has contributed to the factor. The basic statistic used in factor analysis is the correlation coefficient, which determines the relationship between two variables and uses matrix algebra calculated by computing. For all pairs *X_i_*, we want to find factors such that when they are extracted, there is an absence of partial correlation between the tests, that is, the partial correlations are zero [37,38].

In matrix notation, factor analysis can be described by the equation $R=PCP^{\prime }+U^{2}$, where R is the matrix of correlation coefficients among observed variables, *P* is the primary factor pattern or loading matrix (*P*′ is the transpose), *C* is the matrix of correlations among common factors, and *U*^2^ is the diagonal matrix or unique variances [37,38,39]. All statistical work was done using IBM^®^ SPSS^®^ Statistics for Windows, Version 23.0. Armonk, NY: IBM Corp [40].

## 3. Results

### 3.1. Community Health Care Services and Invested Resources

This study compiled a total of 59 care services prevalent in Taiwan and foreign countries. Resources invested in these care services were categorized as follows: (1) a designated space or venue for regular gatherings (hereafter referred to as designated space); (2) materials that cannot be exchanged for other resources after they are provided; (3) monetary resources that can be used to purchase materials; (4) human resources that contain at least three people; (5) expertise required to organize related activities; (6) professional equipment required in related activities; (7) patience needed to participate in activities that last for more than 1 h; and (8) empathy that enables participants to cope with undesirable situations (e.g., odors and mental disorders of service recipients) [7,8]. Therefore, each care service corresponds to at least one resource. For example, sheltered farms (Service 1) that provide jobs for homeless people require eight resources, whereas employment assistance (Service 12) only requires one resource (i.e., expertise). Care services and their required resources are presented in Table 2. However, these 59 kinds of care services were not applicable in every community. In the future, university students can choose service items through the screening mechanism detected from this research. However, the venue, culture, and basic equipment of the implementation targets ought to be considered in future studies, such as network, power supply, etc. Thereafter, comprehensive evaluation should be involved to assess which ones are the most appropriate service activities. This is, therefore, also a limitation of this study due to locality.

### 3.2. Using Factor Analysis to Categorize Characteristics

Initially, matrix calculation was performed to determine the correlations between the resources invested in the 59 care services. The correlation analysis involved 59 samples, each having eight variables (Table 3). As shown in Table 2, except for human resources and patience, which attained a significant correlation coefficient of 0.73, other variables were slightly correlated when analyzed in pairs. This indicated that using each variable to independently examine the care services was infeasible to determine the main resources required by the services. Subsequently, the results of the Kaiser–Meyer–Olkin (KMO) test revealed a KMO index of 0.516 (>0.5), implying the existence of latent principal factors [37,38,39,40]. Therefore, factor analysis was performed to reduce the number of variables and compile the principal factor components.

This study calculated the total amount of explained variance and extracted factors from the data (Table 4). Examining the eigenvalues (>1) of the unrotated component loadings showed that the first three components explained 67.99% of the data. In particular, the first component explained the greatest amount of variance (27.238%), and the second and third components explained 22.325% and 18.429% of the variance, respectively. Analyzing the eigenvalues (>1) of the rotated component loadings revealed that the first three components also explained 67.99% of the data. Particularly, the first component accounted for the greatest amount of variance (25.036%), and the second and third components explained 21.809% and 21.147% of the variance, respectively. In the latter case, although differences between the variances explained were reduced, the total amount of variance explained did not increase. Subsequently, a scree plot was adopted to determine the number of extracted factors. This plot involves a scatter plot that presents factor number on the *x*-axis and unrotated eigenvalues on the *y*-axis [37,38,39,40]. By observing this plot, researchers can subjectively determine a threshold value for factor loading. Eigenvalues vary greatly above the threshold value but slightly below the threshold value. The factor number corresponding to the threshold value denotes the number of factors to be extracted. Adopting a threshold of eigenvalue >1 in the scree plot is reasonable; therefore, three factors were extracted in this study [38].

### 3.3. Factor Naming

Table 5 presents the component loadings of the factors. In the table, human resources and patience were extracted as the variables of the first factor. Designated space and materials were extracted as the variables of the second factor. Expertise and professional devices were extracted as the variables of the third factor [37,38,39].

The first factor was named labor services. This type of care service refers to labor intensive activities that provide care services. The second factor was named high-resource services, which require substantial money and appeal and provide a large amount of materials to service recipients. The third factor was named professional services. This type of service requires expertise, provides expertise required for related activities, and is delivered in various ways.

According to the aforementioned analysis results, community health care services suitable for university students to provide include labor services and professional services. After discussing with the director-general of the community development association, we selected Services 50 (filming community documentaries), 55 (offering community health dance classes), and 37 (archiving community cultural artifacts in databases) as the actual services provided to the Lunfeng community.

## 4. Discussion

### 4.1. Filming a Community Documentary

During the service, the participating university students outlined the filming plan, conducted fieldwork, cultivated their filming skills, learned to utilize storyboards for filming, wrote a script, and practiced film editing techniques. In addition to theories taught in classes, the students acquired valuable experiences when they explored touching true stories, witnessed the ordinary happiness reflected by these stories, and filmed a documentary integrating the local cultural landscapes and histories [32,33].

First, the students sought community stories for the film. They adopted the sugar factory in the community as the main theme of the stories because the sugar factory had long been crucial to the community’s industrial development before it was shut down. In addition, the factory was a critical source of income for multiple local families. Moreover, the childhood memories commonly shared by older adults in the community are closely related to the sugar factory. Accordingly, the stories of the sugar story were used as the filming materials. Initially, the story setting was designed to focus on the history of the sugar industry associated with the sugar factory when it was still in operation. However, this setting was slightly relevant to the community. Therefore, the students revised the filming plan, sought community residents who had worked (either part time or full time) at and retired from the sugar factory, and collected their stories through fieldwork. Subsequently, the story outline and subsections were determined. During data collection, the students found that the prosperity of the sugar factory had impeded the interactions between ordinary community children and factory employees’ children because the factory dormitory was walled off. In addition, the factory paid particular attention to providing benefits for its employees. For example, the provision of a public bathhouse, an old television in the Zhongshan Hall, and train-fare concessions had excited envy from local residents who were unrelated to the factory employees. Furthermore, interviews were administered to the children of the employees in the Special Police Corps. Overall, 14 local residents were interviewed to complete the entire film.

### 4.2. Offering Community Health Dance Classes

This study designed a dancing exercise program suitable for older adults. The dance was composed of simple and repetitive movements designed at a level of difficulty for kindergarten children; hence, older adults should have no problem performing it [41]. The dancing music spanned 8 minutes. The participants of this activity comprised nine older adults and six community residents, who practiced 2 h daily. During the practice, taping marks were placed on the ground for instructing the positions of the participants. Subsequently, the taping marks were removed to examine how the participants performed with the marks. After a week of intensive practice, the participants performed in a large event held by Taiwanese government. The performance was well received, and the participants gained confidence through this activity. Therefore, this care service exerted a positive effect on the community development.

### 4.3. Archiving Community Cultural Artifacts in Databases

In 2014, the Lunfeng community established a cultural living museum that achieved and organized the community’s meaningful cultural artifacts. The archiving process revealed the distribution of cultural artifacts collected from the Lunfeng community. Specifically, the artifacts constituted 11 production tools (41%), 4 cooking utensils (15%), 6 household tools (22%), 2 transportation-related objects (7%), and 4 musical instruments (15%). The statistics revealed that production tools accounted for the highest number of the collected and intact artifacts, indicating that the community mainly focuses on agricultural production. Household artifacts accounted for the second largest category, and musical instruments and cooking utensils each accounted for 15% of the collection, indicating that the residents also pursue various hobbies to entertain themselves in leisure time [42].

## 5. Conclusions

This study pointed out that postmodern community management pertains to an autonomous managerial mechanism. It requires broad-minded thinking, dialogue, problem-solving skills, and cooperation to shape the context of community relationships. During this period (2015–2020), university students were recruited to deliver community health care services selected in this study (i.e., filming community documentaries, offering community health dance classes, and archiving community cultural artifacts in databases). Moreover, interviews were administered to facilitate the implementation of the actual services. The results showed that three principal components occurred, namely labor services, high-resource services, and professional services, for a total explanatory power of 67.99%. The individual explanatory power of these components accounted for 25.04%, 21.81%, and 21.15%, respectively. After factor extractions, three factor strata were determined and individually named as labor services, high-resource services, and professional services. This study adopted a qualitative method involving audio/video recording and adopted photo-voice and photo elicitation. Community health care services suitable for university students were selected using a multivariate method. After the filming the community documentary and teaching the health dance program, the participating students established a friendly relationship with the local residents, indicating the favorable effect of the community health care services performed in this study. In addition, the establishment of community cultural artifact databases rendered the overall care services more feasible for continuation. These care services enabled the university students to experience social concerns that were otherwise unobservable in academic theories. Overall, these activities have both theoretical and practical implications.

## Figures and Tables

**Table 1:** Basic information of population in the study area.

	Male	Age	Female
**Age Distribution**	99 (7.0%)	0–9	113 (7.6%)
	130 (9.1%)	10–19	134 (9.0%)
	195 (13.7%)	20–29	196 (13.2%)
	213 (15.0%)	30–39	210 (14.1%)
	219 (15.4%)	40–49	212 (14.2%)
	222 (15.6%)	50–59	211 (14.1%)
	189 (13.3%)	60–69	203 (13.6%)
	154 (10.8%)	>70	214 (14.3%)
Male	1419	-	-
Female	1493	-	-
Totals	2912	-	-
Number of Households	1107	-	-

(Information updated: June 2020).

**Table 2:** Common community health care services in Taiwan and abroad.

1. Sheltered Farms that Provide Jobs for Homeless People (1)(2)(3)(4)(5)(6)(7)(8)	21. Building Friendly Environments (2)(4)(5)	41. Providing New and Reliable Health Knowledge (5)
2. Reviving regular community activities (2)(3)(5)	22. Physical fitness services (2)(4)(5)(6)(7)	42. Promoting social participation (2)(3)(4)(7)
3. Repairing summer camp facilities for disabled children (2)(3)(4)(5)(6)(7)(8)	23. Reminiscence therapy (4)(5)(7)	43. Painting the shelters of homeless people or abused women (2)(4)(7)
4. Building new community facilities for the needed (1)(2)(3)(4)(5)(6)(7)	24. Art therapy (4)(5)(7)	44. Painting/maintaining public parks or playgrounds (2)(4)(7)
5. Organizing special festival activities for the community (2)(3)(4)(5)(6)(7)	25. Community seminars (4)(5)	45. Charity shops collecting and selling secondhand goods (1)(2)
6. Improving schools (1)(2)(3)(4)(5)(6)(7)	26. Medical counseling (4)(5)(6)(8)	46. Cleaning the environment (4)(7)(8)
7. Enhancing community health care network (3)(5)	27. Spiritual care (religious belief) (5)	47. Care visits (2)(4)(7)(8)
8. Providing sufficient and sound computer equipment (1)(2)(5)	28. Promoting dementia prevention policies (4)(5)(7)(8)	48. Support services for disabled people who lead an independent life (4)(7)(8)
9. Organizing arts nights (1)(2)(4)(5)(6)	29. Home economics education (1)(4)(5)	49. Study groups (1)(2)(4)(7)
10. Service centers for foreign spouses (1)(2)(5)(7)	30. Health education (5)(8)	50. Filming community documentaries (4)(5)(7)
11. Recycling (2)(4)(5)(7)(8)	31. Teaching foreign languages (5)	51. Providing sports and entertainment facilities (1)(2)
12. Employment assistance (5)	32. Providing instructions on how to use the internet (5)	52. Establishing community libraries (1)(2)(4)(7)
13. Dance performances (4)(5)(7)	33. Encouraging lifelong learning (4)(5)(7)	53. Food provision (2)(4)(7)
14. Pottery DIY courses (1)(5)(6)	34. Health seminars (5)(8)	54. Shower provision (2)(4)
15. Painting DIY courses (1)(5)(6)	35. Spiritual restoration for homeless people (4)(5)(7)	55. Offering community health dance classes (4)(5)(7)
16. Free clinics (4)(5)(6)(7)	36. Promoting breast cancer prevention in the community (5)	56. Lending assistive devices (e.g., wheelchairs, hospital beds, crutches, and walkers) (1)(2)
17. Free haircuts (4)(5)(6)(7)	37. Archiving community cultural artifacts in databases (4)(5)(7)	57. Providing open spaces (1)
18. Providing psychological counseling services (5)(7)(8)	38. Promoting preventive health care (5)	58. Providing storage rooms (1)
19. Health-promoting services (4)(5)(6)(7)(8)	39. Advocating healthy diets (5)	59. Phone greetings (4)(7)
20. Massage services (4)(5)(7)(8)	40. Publicizing appropriate concepts of medication and seeking medical advice (5)	-

Note: Resources required by each service are numbered as follows: (1) designated space; (2) materials; (3) monetary resources; (4) human resources; (5) expertise; (6) professional equipment; (7) patience; and (8) empathy. Whether it is executable still needs to be considered on site.

**Table 3:** Correlation matrix of service resources required.

Item	Item (1)	Item (2)	Item (3)	Item (4)	Item (5)	Item (6)	Item (7)	Item (8)
Item (1)	1.00	0.37 *	0.09	−0.22 *	−0.23 *	0.23 *	−0.20	−0.23 *
Item (2)	-	1.00	0.39 **	0.21	−0.37 *	0.16	0.18	−0.09
Item (3)	-	-	1.00	0.11	0.13	0.39 **	0.17	0.03
Item (4)	-	-	-	1.00	−0.10	0.26 *	0.73 ***	0.17
Item (5)	-	-	-	-	1.00	0.32 **	−0.10	0.05
Item (6)	-	-	-	-	-	1.00	0.16	0.11
Item (7)	-	-	-	-	-	-	1.00	0.24 *
Item (8)	-	-	-	-	-	-	-	1.00

Note: Resources required by each service are numbered as follows: (1) designated space; (2) materials; (3) monetary resources; (4) human resources; (5) expertise; (6) professional equipment; (7) patience; and (8) empathy. Single tailed test. *p* Value < 0.05 *, *p* Value < 0.01 **, *p* Value < 0.001 ***; Kaiser–Meyer–Olkin Measure of Sampling Adequacy (KMO) is 0.516; Bartlett’s Test of Sphericity is very significant *p* < 0.001.

**Table 4:** Total variance analysis and factors loading.

	Initial Eigenvalues			Extraction Sums of Squared Loadings			Rotation Sums of Squared Loadings		
Factors	Total	% of Variance	Cumulative (%)	Total	% of Variance	Cumulative (%)	Total	% of Variance	Cumulative (%)
1	2.18	27.24	27.24	2.18	27.24	27.24	2.00	25.04	25.04
2	1.79	22.33	49.56	1.79	22.33	49.56	1.75	21.81	46.85
3	1.47	18.43	67.99	1.47	18.43	67.99	1.69	21.15	67.99
4	0.84	10.54	78.53	-	-	-	-	-	-
5	0.76	9.46	87.99	-	-	-	-	-	-
6	0.38	4.79	92.78	-	-	-	-	-	-
7	0.36	4.43	97.21	-	-	-	-	-	-
8	0.22	2.79	100.00	-	-	-	-	-	-

Note: extraction method: principal component analysis.

**Table 5:** Component loading.

Resources	Component		
	1	2	3
Human Resources	* 0.803	−0.272	−0.254
Patience	* 0.793	−0.301	−0.283
Monetary Resources	0.526	0.331	0.445
A Designated Space or Venue	–0.032	* 0.811	0.077
Materials	0.499	* 0.681	−0.204
Empathy	0.309	–0.459	0.027
Expertise	−0.069	−0.393	* 0.808
Professional Equipment	0.528	0.160	* 0.656

Note: 3 components extracted; extraction method: principal component analysis. The bold word with * is main construction variable.
